# Early Administration of the Phytocannabinoid Cannabidivarin Prevents the Neurobehavioral Abnormalities Associated with the *Fmr1*-KO Mouse Model of Fragile X Syndrome

**DOI:** 10.3390/cells12151927

**Published:** 2023-07-25

**Authors:** Marika Premoli, William Fyke, Luigi Bellocchio, Valerie Lemaire, Marie Wolley-Roberts, Bruno Bontempi, Susanna Pietropaolo

**Affiliations:** 1 CNRS, EPHE, INCIA, UMR 5287, Univ. Bordeaux, 33000 Bordeaux, France; 2 Department of Molecular and Translational Medicine, University of Brescia, 25123 Brescia, Italy; 3 Graduate Program in Neural and Behavioral Science, SUNY Downstate Medical Center, 450 Clarkson Avenue, Brooklyn, NY 11203, USA; 4 INSERM, U1215 NeuroCentre Magendie, Group Endocannabinoids and Neuroadaptation, University of Bordeaux, 33077 Bordeaux, France; 5 Jazz Pharmaceuticals, Inc., Cambridge OX4 2RW, UK

**Keywords:** phytocannabinoids, *Fmr1*, mouse behavior, neurodevelopmental disorders, interleukins, neurotrophins

## Abstract

Phytocannabinoids, including the non-addictive cannabis component cannabidivarin (CBDV), have been reported to hold therapeutic potential in several neurodevelopmental disorders (NDDs). Nonetheless, the therapeutic value of phytocannabinoids for treating Fragile X syndrome (FXS), a major NDD, remains unexplored. Here, we characterized the neurobehavioral effects of CBDV at doses of 20 or 100 mg/kg in the *Fmr1*-knockout (*Fmr1*-KO) mouse model of FXS using two temporally different intraperitoneal regimens: subchronic 10-day delivery during adulthood (Study 1: rescue treatment) or chronic 5-week delivery at adolescence (Study 2: preventive treatment). Behavioral tests assessing FXS-like abnormalities included anxiety, locomotor, cognitive, social and sensory alterations. Expression of inflammatory and plasticity markers was investigated in the hippocampus and prefrontal cortex. When administered during adulthood (Study 1), the effects of CBDV were marginal, rescuing at the lower dose only the acoustic hyper-responsiveness of *Fmr1*-KO mice and at both doses their altered hippocampal expression of neurotrophins. When administered during adolescence (Study 2), CBDV at both doses prevented the cognitive, social and acoustic alterations of adult *Fmr1*-KO mice and modified the expression of several inflammatory brain markers in both wild-type littermates and mutants. These findings warrant the therapeutic potential of CBDV for preventing neurobehavioral alterations associated with FXS, highlighting the relevance of its early administration.

## 1. Introduction

Recent lines of evidence have highlighted the relevance of the endocannabinoid system (ECS) as an important modulator of neuronal functions and a promising therapeutic target for treating a variety of psychiatric disorders [1]. In the brain, the ECS comprises CB1 and CB2 cannabinoid receptors, their endogenous lipid ligands referred to as endocannabinoids (ECBs) and the enzymatic machinery involved in the synthesis and degradation of ECBs. The neurofunctional relevance of this signaling system is well acknowledged, as demonstrated by the abundance of CB1 receptors throughout the brain relative to CB2 receptors whose lower expression is mainly restricted to microglia and vascular components [2]. Furthermore, CB1 receptors, generally predominantly expressed presynaptically, modulate brain functions via the direct control of the release of several neurotransmitters including GABA, glutamate and serotonin. The ECS also regulates synaptogenesis and neuronal interconnectivity during development [3,4,5], two processes whose defects have been reported as key determinants in major neurodevelopmental disorders (NDDs). Hence, the role of the ECS in NDDs has attracted considerable interest (e.g., [6,7,8]), particularly as concerns its implication in the pathophysiology of Fragile X Syndrome (FXS) [9,10,11,12].

FXS is a rare NDD due to an unstable expansion of CGG repeats in the X-linked gene *FMR1*, producing loss of FMRP, a synaptically expressed RNA-binding protein regulating protein synthesis [13,14]. FXS is considered the most common monogenic cause of inherited intellectual disability and autism. Building upon the widely used animal model of FXS, i.e., the *Fmr1*-knockout (*Fmr1*-KO) mouse, several studies have suggested a major role of the ECS in the pathogenesis of this disease. First, FXS is characterized by an uncontrolled activity of the metabotropic glutamate receptor 5 [15,16] and abnormal mammalian target of rapamycin (mTOR) signaling [17,18,19], two pathways that are heavily involved in ECS functionality [20,21,22,23]. Second, previous findings [9,10,11,12], including our own [24], have revealed an aberrant hyperfunctionality of the ECS in *Fmr1*-KO mice, highlighting the possibility that behavioral deficits may result from dysfunctional CB1-mediated signaling in this mouse model. Accordingly, downregulating the ECS as a therapeutic strategy in *Fmr1*-KO mice has yielded encouraging results [9,25]. Within this pharmacological framework, cannabidiol (CBD) and its analogs can modulate the functionality of the ECS by inhibiting the enzymes involved in the synthesis of endocannabinoids [26]. Importantly, these phytocannabinoids do not directly act on the major cannabinoid receptor CB1, thus avoiding the heavy undesirable side effects of CB1 antagonists, for instance rimonabant, as well as the noxious psychotropic and addictive effects of other cannabinoids, such as the most naturally abundant and best known Delta^9^-tetrahydrocannabinol (THC). Hence, these non-psychoactive phytocannabinoids hold promises for treating FXS as they could attenuate the ECS hyperfunctionality observed in FXS without the burden of major side effects. The therapeutic applications of these compounds have already been explored in a variety of pathologies [27,28,29,30,31,32]. In particular, pre-clinical studies in rodents have shown that cannabidivarin (CBDV), a propyl analog of CBD, can induce anti-convulsant [33] and anxiolytic effects [34], together with powerful anti-inflammatory properties [28]. CBDV has also been reported to rescue some of the neurobehavioral alterations found in animal models of Rett syndrome [6,7] and autism spectrum disorder (ASD; [35]). However, the therapeutic potential of CBDV for treating FXS has not been investigated yet.

**Figure 1:**
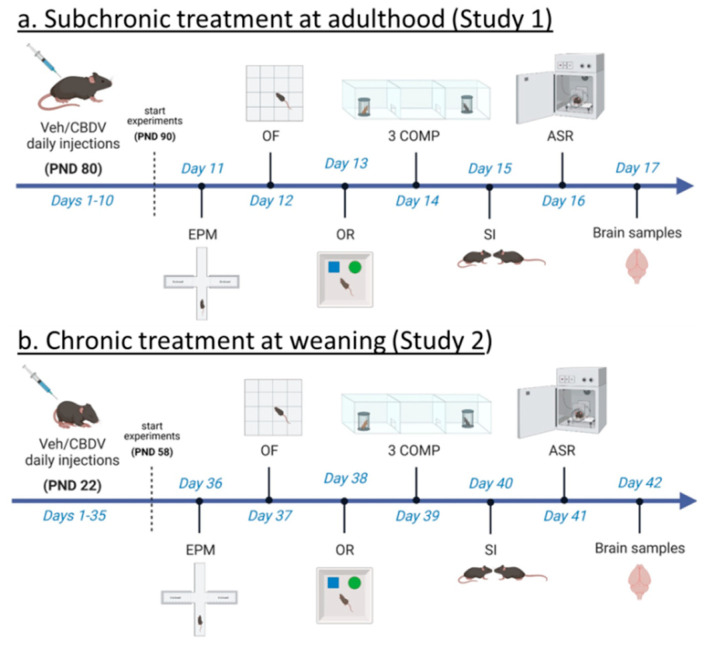
Experimental timeline and procedures used in Study 1 and Study 2. For Study 1 (**a**), treatment administration started at adulthood (i.e., approximately 3 months of age) and 10 days before the beginning of behavioral testing. Daily intraperitoneal injections of vehicle (Veh) or cannabidivarin (CBDV) solutions (20 or 100 mg/kg) were given during the entire experimental period, including the days of behavioral testing and brain sampling (one hour before their beginning). For Study 2 (**b**), daily intraperitoneal injections started the day after weaning (i.e., at 3 weeks of age) and 5 weeks before the beginning of behavioral tests. Injections were continued during the entire experimental period, including the days of behavioral testing during which they were administered after completion of each testing procedure. EPM = elevated plus maze, OF = open field, OR = object recognition, 3 COMP = three-compartment test, SI = social interaction, ASR = acoustic startle response. PND = post-natal day.

Here, we aimed at evaluating the therapeutic impact of CBDV in the *Fmr1*-KO mouse model of FXS by combining two experimental approaches (as schematized in Figure 1). In the first study (Study 1), we evaluated whether FXS-like neurobehavioral phenotypes could be rescued by a sub-chronic (10-day) CBDV treatment that started at adulthood, i.e., once the pathology is fully expressed in the *Fmr1*-KO mouse model [36]. The goal of the second study (Study 2) was instead to investigate whether the therapeutic effects of CBDV could somehow be enhanced by administering the CBDV treatment preventively at weaning, a developmental stage with high levels of neurobehavioral plasticity [37]. Our hypothesis was that juvenile chronic (during 5 weeks) CBDV administration would be more efficacious than an adult sub-chronic treatment and could prevent the expression of the neurobehavioral abnormalities typically observed in adult *Fmr1*-KO mice. Both studies used the doses of 20 and 100 mg/kg, as in previous research using CBDV in animal models of other NDDs, i.e., Rett syndrome [6,7] and ASD [35]. In the two studies, *Fmr1*-KO mice and their littermates underwent a comprehensive test battery tailored to several behavioral domains that we and others previously reported to be robustly altered in this mouse model of FXS [38,39,40,41,42]. Accordingly, behavioral screening included paradigms taxing motor activity in the open field, object recognition memory, social interest (in the three-compartment and direct social interaction tests) and sensory processing using acoustic startle. Although not consistently described in *Fmr1*-KO mice, emotional alterations in the elevated plus maze were also evaluated in order to assess potential confounding differences in anxiety-like behavior induced by CBDV treatments. At the end of both studies, brain samples were collected from tested mice in order to investigate the ability of CBDV to reverse (Study 1) or prevent (Study 2) the altered expression patterns of inflammatory and plasticity markers in cortex and hippocampus, two brain regions where the FMRP protein is most abundant [43,44] and in which expression of these markers has been reported to be altered in *Fmr1*-KO mice [40]. Also, the brain expression of some of these inflammatory markers (e.g., TNFα, CD11b) was previously shown to be modulated by CBDV, at least in other animal models of developmental pathologies [35].

## 2. Materials and Methods

### 2.1. Animals

Subjects were male C57BL/6J (B6) *Fmr1*-KO mice and their wild-type (WT) littermates, bred in our animal facility at Bordeaux University for more than 10 generations. Breeding trios were formed by mating two heterozygous *Fmr1* females with a wild-type B6 male purchased from Janvier (Le Genest St Isle, France). After 2 weeks, the sire was removed and females were single caged and left undisturbed until post-natal day (PND) 8 of the pups. On this same day, pups were marked with paw tattoos using a non-toxic ink (Ketchum permanent Tattoo Inks green paste, Ketchum MFG Co, New York, NY, USA), and tail samples were collected for PCR assessment of the genotypes as previously described [45]. Mice were weaned at PND 21 and group-housed with their same-sex littermates (3–5/cage). Only male mice were used for the study, as they are the most commonly employed in mouse studies on FXS due to the higher prevalence of this syndrome in the male sex [36]. Only litters including males of both genotypes (WT and KO) were used for all experiments.

NMRI female mice (12 ± 2 week-old) and juvenile (4 week-old) males purchased from Janvier (Le Genest St Isle, France) were used as social stimuli during the direct social interaction and three-compartment tests, respectively. This strain has been selected for its high level of sociability [46] and was previously employed in several social studies on *Fmr1*-KO mice [40,41,42]. Mice were group-housed (4–5 cage) and left undisturbed upon arrival for one week before the social interaction test.

All animals were housed in polycarbonate standard cages (33 × 15 × 14 cm in size; Tecniplast, Limonest, France), provided with litter (SAFE, Augy, France) and a stainless-steel wired lid. Food (SAFE, Augy, France) and water were provided ad libitum. The animals were maintained in a temperature (22 ± 1 °C) and humidity (55 ± 10%) controlled vivarium, under a 12:12 h light–dark cycle (lights on at 7 a.m.). All experimental procedures were in accordance with the European Communities Council Directive of 24 November 1986 (86/609/EEC) and local French legislation (Authorization N° 2017073113175079).

### 2.2. Experimental Procedures 

#### 2.2.1. CBDV Administration at Adulthood (Study 1)

In Study 1, adult (12 ± 1 weeks of age) mice of both genotypes were assigned to one of the three following experimental conditions: vehicle (VEH: Cremophor^®^ EL:Ethanol:saline in a ratio of 1:2:17), CBDV at a dose of 20 mg/kg (CBDV-20) or CBDV at a dose of 100 mg/kg (CBDV-100). CBDV (synthetic; purity by HPLC > 99%) was supplied by GW Research Limited (now part of Jazz Pharmaceuticals, Inc., Cambridge, UK) and stored at approximately −20 °C, protected from light. Injectable solutions were prepared fresh each day and were continuously stirred until injection. A total of 46 adult males were used as subjects in Study 1 (*n* = 8 for WT-VEH, KO-VEH, WT-CBDV-20 and KO-CBDV-100 groups; *n* = 7 for *Fmr1*-KO-CBDV-20 and WT-CBDV-100 groups). The number of subjects per experimental conditions was similar to that used in previous studies showing therapeutic effects of phytocannabinoids in other animal models (e.g., [6,7]).

*Fmr1*-KO mice and WT littermate controls were injected intraperitoneally (10 mL/kg), once a day around 9.00 a.m., during the entire duration of the study, i.e., for 17 consecutive days. Behavioral tests began after 10 days of injections, according to the timing described in Figure 1a. The order of the tests was based on the need of performing first the tests that are the most sensitive to previous testing experience (such as the elevated plus maze), while leaving for last those involving a higher degree of stressful experience (such as the acoustic startle which requires a short confinement in the startle box). A similar sequence of behavioral tests was employed in several previous studies on *Fmr1*-KO mice (e.g., [40,41,47,48]). All procedures were performed during the light phase, between 9 a.m. and 5 p.m. Mice assigned to each of the three experimental conditions were injected one hour before the beginning of each testing procedure; after injection, each mouse was left undisturbed in a waiting cage containing sawdust bedding, food and water. Mice were habituated to the testing room at least 90 min before the beginning of each behavioral test.

#### 2.2.2. CBDV Administration at Weaning (Study 2)

In Study 2, the day after weaning (i.e., on PND 22), male mice of both genotypes were assigned to one of the three following experimental conditions, VEH, CBDV-20 or CBDV-100. All solutions were prepared as described for Study 1. A total of 47 mice were subjected to all behavioral tests and brain analysis (*n* = 7 for WT-VEH and WT-CBDV-100 groups; *n* = 8 for WT-CBDV-20, KO-CBDV-20 and KO-CBDV-100 groups; *n* = 9 for the KO-VEH group). *Fmr1*-KO mice and WT littermates were injected intraperitoneally once a day during the entire duration of the study (Figure 1b). Behavioral tests began after 5 weeks of injections, following the timing described in Figure 1b and using the same test sequence as for Study 1. Body weight gain was assessed by comparing the animals’ body weight before and after the 5 weeks of treatment in order to evaluate potential group differences in the body growth occurring from weaning to adulthood.

All procedures were performed during the light phase, as in Study 1. On testing days, the treatments were given after the end of each behavioral test, approximately 24 h from behavioral and brain assessment to minimize acute effects of CBDV. The order of testing was counterbalanced across experimental groups, and mice were habituated to the testing room before behavioral testing as described for Study 1.

#### 2.2.3. Behavioral Assessment (Studies 1 and 2)

*Elevated plus maze*. The apparatus and procedures were described in detail elsewhere [47,49]. Briefly, each mouse was gently placed in the center of the elevated plus maze and left free to explore the maze during 5 min. Videos recorded from a digital camera above the maze were analyzed manually by an observer blind to the experimental condition of the animals using Observer XT (Version 7, Noldus Technology, Wageningen, The Netherlands). Anxiety-like behaviors were measured as follows: percent time in open arms = time in open arms/time in all arms × 100. Locomotor activity was assessed by scoring the total number of entries into the arms of the maze.

*Open field (habituation phase of object recognition test)*. The apparatus consisted of 2 identical plastic 24 × 30 cm rectangular arenas surrounded by 22 cm-high walls. The arenas were located in a testing room under diffused dim lighting (30 lux in the arena center). A digital camera was mounted directly above the arenas, capturing images at 5 Hz that were transmitted to a PC running the Ethovision tracking system (version 11, Noldus, Wageningen, The Netherlands). Each mouse was gently placed in the center of the appropriate arena and allowed to explore it undisturbed for 20 min. The choice of the arena was counterbalanced across experimental groups. Locomotor activity was indexed by the total distance traveled. Anxiety was assessed by calculating the percentage of time spent in the center of the arena.

*Object recognition*. The open field test served as the habituation phase for the object recognition test that was described in detail elsewhere [40]. Briefly, at the end of the open field session, two identical objects were placed in two opposite corners, and the mouse was introduced in the center of the arena for a 5 min sample phase. Twenty-four hours later, the mouse was returned to the arena for a 5 min test phase where one of the objects was replaced by a novel one of different shape and material. Both the type of object used for the sample phase and the position of the novel object during the test phase were counterbalanced across experimental groups. During the training and test phases, the time spent sniffing each object was manually scored by an observer unaware of the experimental conditions of the animals using Observer XT (version 7, Noldus, Wageningen, The Netherlands). To measure object recognition during the test phase, a percent recognition index was calculated using the following formula: T_novel object_/(T_novel object_ + T_familiar object_) × 100 (T: time). The lack of novel object recognition was set at 50%. At the end of the sample and test phases, the apparatus as well as the objects were cleansed with a 30% ethanol solution and dried.

*Three-compartment test*. The apparatus (described in detail elsewhere [41]) consisted of a central chamber connected on each side to another compartment containing a perforated stimulus cage (8 × 8 × 15 cm) to allow the test mouse to interact with the mouse or the object inside the stimulus cage. The object employed for the test was a plastic black cylinder, and the stimulus mice were NMRI juvenile males, in order to minimize aggressive tendencies and exclude sexual interest. Each experimental subject was introduced in the middle of the central compartment and allowed to explore the apparatus for 3 trials of 5 min each. In trial 1, habituation to the apparatus containing empty stimulus cages was evaluated, while in trial 2, the preferential exploration of the social (a juvenile male mouse) versus the non-social (an object) novel stimulus was measured. In trial 3, the preferential exploration of a novel versus familiar social stimulus was assessed by replacing the object with a novel stimulus mouse.

In all trials, the total distance traveled as well as the time spent in each contact area (20 × 22 cm) containing the stimulus cages was computed using the Ethovision tracking system (version 11, Noldus, The Netherlands). A percentage score was also computed for the last two trials as follows:

In trial 2: Sociability index = T_social stimulus_/(T_social stimulus_ + T_non-social stimulus_) × 100.

In trial 3: Social novelty preference index = T_novel social stimulus_/(T_novel social stimulus_ + T_familiar social stimulus_) × 100.

At the end of each trial, the experimental animal was confined for 30 s in the central compartment by means of two Plexiglas magnetic doors. At the end of the third trial, the apparatus as well as the object and the stimulus cages were cleansed with a 30% ethanol solution and dried.

*Direct social interaction*. Direct social interaction was assessed as described in detail elsewhere [41]. Briefly, an unfamiliar adult NMRI female mouse was introduced into a testing cage (32 × 14 × 12.5 cm, with a flat metal grid as cover and approximately 3 cm of clean sawdust bedding) to which experimental subjects were habituated for one hour. Six min testing sessions were recorded, and videos were analyzed with an Observer XT (version 7, Noldus, Wageningen, The Netherlands). One observer who was unaware of the experimental conditions of the animals scored the time spent performing affiliative behaviors, i.e., social investigation (nose, body and anogenital sniffing) and contacts. At the beginning of the testing day, the estrous cycle of the stimulus females was assessed through the analysis of the vaginal smear, so that only females in the non-estrous phase were used for social interaction sessions.

*Sensory responsiveness (acoustic startle test)*. The apparatus consisted of four acoustic startle chambers for mice (SR-LAB, San Diego Instruments, San Diego, CA, USA) described in detail elsewhere [42,47]. Twenty-four hours before testing, mice were placed in the startle chamber for 5 min without being exposed to any stimuli in order to habituate them to the confinement and reduce the related stress. On the test day, after a 5 min habituation period with continuous white noise of 66 dB (background), mice were presented with pulses of 20 ms duration and of varying intensity: +6, +12, +18 and +24 dB over background levels (namely 72, 78, 84 and 90 dB), as previously described in detail [42,47]. Vibrations of the Plexiglas enclosure caused by the whole-body startle response of the animal were converted into analogue signals, digitized and stored by a computer.

#### 2.2.4. Brain Assessment of Inflammatory and Plasticity Markers through RT-qPCR (Studies 1 and 2)

Mice were sacrificed by cervical dislocation. Brains were immediately extracted and cut into two hemispheres that were separately frozen using dry ice. Only one hemisphere underwent RNA extraction while the other was stored at −80 °C as backup. Whole brain tissue was sectioned using a Leica cryostat to generate 50 μm-thick sections. Sections were collected on polyethylene-naphthalate membrane 1 mm glass slides (series of 8–10 sections per slide) that were pretreated with heat to inactivate RNases. Series corresponding to the prefrontal cortex (PFC) (infralimbic and prelimbic areas) were collected from bregma +1.98 mm to +1.54 mm according to the Paxinos mouse stereotaxic atlas. Series corresponding to Cornu Ammonis 1 (CA1), cornu Ammonis 3 (CA3) and dentate gyrus (DG) of the hippocampus were collected from bregma −1.22 mm to −2.80 mm. To enable cell identification, sections were then stained with cresyl violet using a protocol compatible with subsequent RNA isolation. RNase-free water and solutions were used for all steps. Sections were fixed for 30 s with 95% ethanol, followed by 75% ethanol for 30 s and by 50% ethanol for 30 s to remove the OCT tissue freezing medium compound used during cryostat sectioning to provide a specimen matrix. Sections were subsequently stained with 1% cresyl violet in 50% ethanol for 30 s and dehydrated in 50%, 75% and 95% ethanol for 30 s each, and 2 times in 100% ethanol for 30 s. Stained slides were stored at −80 °C until laser capture microdissection.

Laser Pressure Catapulting microdissection (LPC) of samples was performed using a PALM MicroBeam microdissection system version 4.6 equipped with the PALM RoboSoftware (P.A.L.M. Microlaser Technologies AG, Bernried, Germany). Laser power and duration were adjusted to optimize capture efficiency. Microdissection was performed at 5X magnification. The microdissections of pure brain structures were collected in adhesives caps and re-suspended in 250 µL guanidine isothiocyanate-containing buffer (BL buffer from ReliaPrep™ RNA Cell Miniprep System, Promega, Madison, WI, USA) with 10 µL 1-Thioglycerol and stored at −80 °C until extraction was completed. Total RNA was extracted from microdissected tissues using the ReliaPrep™ RNA Cell Miniprep System (Promega, Madison, WI, USA) according to the manufacturer’s protocol. The integrity of the RNA was checked by capillary electrophoresis using the RNA 6000 Pico Labchip kit and the Bioanalyser 2100 (Agilent Technologies, Massy, France), and quantity was estimated using a Nanodrop 1000 (Thermo Scientific, Waltham, MA, USA). RNA integrity numbers (RIN) were above 7/8.

RNA was processed and analyzed according to an adaptation of published methods [50]. Briefly, cDNA was synthesized from 140 ng of total RNA for each structure by using qSript™ cDNA SuperMix (Quanta Biosciences, Rockville, MD, USA). qPCR was performed with a LightCycler^®^ 480 Real-Time PCR System (Roche, Meylan, France). qPCR reactions were done in duplicate for each sample by using LightCycler 480 SYBR Green I Master (Roche) in a final volume of 10 μL. The qPCR data were exported and analyzed in an informatics tool (Gene Expression Analysis Software Environment) developed at the University of Bordeaux. The Genorm method was used to determine the reference gene [50]. Relative expression analysis was normalized against two reference genes. Succinate dehydrogenase complex subunit (Sdha) and tubulin alpha 4a (Tuba4a) were used as reference genes for PFC. Succinate dehydrogenase complex subunit (Sdha) and tyrosine 3 mono-oxygenase tryptophan 5 monooxygenase (Ywhaz) were used as reference genes for CA1. Tuba4a and glyceraldehyde-3-phosphate dehydrogenase (Gapdh) were used as reference genes for CA3. Tuba4a and non-POU-domain-containing octamer binding protein (Nono) were used as reference genes for DG. The relative level of expression was calculated with the comparative (2^−ΔΔCT^) method [51]. Primer sequences are reported in Supplementary Table S1.

### 2.3. Statistical Analysis

Behavioral and brain marker data were inspected for the identification of possible outliers using the Grubbs’ test or the extreme studentized deviate ESD method. Identified outliers for a given variable were excluded from the statistical analysis. This explains the slight differences in the total number of animals per group among certain statistical comparisons. For each variable analyzed, the exact sample size is indicated in the corresponding figure or in its legend.

Normality was assessed through the Shapiro–Wilk test for each experimental group and each variable of interest. Data from startle reactivity did not show a normal distribution at all stimulus intensities and were therefore subjected to natural logarithmic (ln) transformation in order to meet the normality requirements of ANOVA. For all other variables, data distribution was found to be normal, and a parametric 2 × 3 ANOVA with genotype and treatment as the between-subject factors was applied. Within-subject factors were included according to the specific test and used as repeated measures in the ANOVA. These included, for example, 5 min-time bins for the total distance traveled in the open field and 3 min-time bins for the social interaction test and the stimulus intensity for the acoustic startle assessment.

Post hoc comparisons were conducted using Tuckey’s HSD test when a significant interaction was found. Otherwise, separate one-way ANOVAs for each treatment group with genotype as the between subject factor were conducted, if appropriate. For the object recognition index, sociability and social novelty scores in the three-compartment test, a one-sample *t*-test was used for comparison with chance level/lack of preference (i.e., 50%) instead of the ANOVA, as performed in previous behavioral studies (see for example, [39,52]). All analyses were carried out using Statview version 5.0.1 (SAS institute Inc., Cary, NC, USA) and PAWS Statistics 18 (SPSS Inc., Chicago, IL, USA).

## 3. Results

### 3.1. Study 1: Effects of Subchronic Administration of CBDV at Adulthood

#### 3.1.1. Behavioral Profiling

- Elevated plus maze

We examined the percent time spent in the open arms of the maze as an index of anxiety-like behavior. A two-way ANOVA revealed no significant effect of genotype (F_1,24_ = 2.77; NS) or CBDV treatment (F_1,24_ = 4.00; NS). *Fmr1*-KO mice and their WT littermates spent a similar amount of time in the open arms, a pattern that was unaffected by CBDV, regardless of the dose (genotype × treatment interaction: F_1,24_ = 2.54; NS). Thus, anxiety levels were similar across groups as shown in Figure S1a. Likewise, locomotor activity did not differ among experimental groups, as demonstrated by a similar number of total arm entries across groups (no effect of genotype (F_1,24_ = 0.96; NS), CBDV treatment (F_1,24_ = 0.22; NS) or their interaction (F_1,24_ = 0.89; NS) (Figure S1b).

- Object recognition test

**Figure 2:**
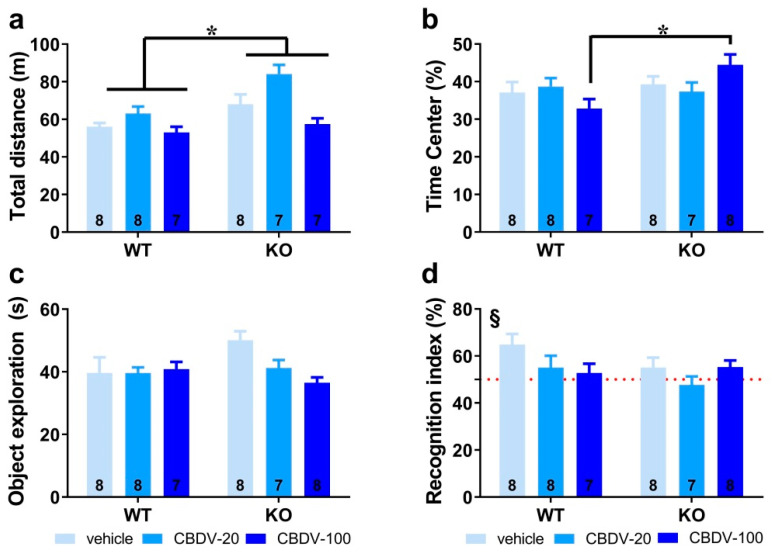
Effects of adult subchronic CBDV administration on object recognition memory (Study 1). Locomotor activity (**a**) and anxiety-like behavior (**b**) were measured during the 20 min habituation session in the empty open field. The time spent exploring both objects (**c**) and the novel object recognition index (**d**) were computed during the 5 min sample and test phases, respectively. All behavioral parameters were measured in WT and *Fmr1*-KO mice treated with vehicle solution, CBDV at the dose of 20 mg/kg (CBDV-20) or 100 mg/kg (CBDV-100). Data are expressed as mean ± SEM. Numbers in histograms indicate sample size for each group. * *p* < 0.05; § versus chance level (50%, red dotted line).

*Habituation phase.* We took advantage of the open field arena used for the habituation phase of the object recognition paradigm to first assess hyperactivity, which is considered a robust end point for *Fmr1*-KO mice. As illustrated in Figure 2a, *Fmr1*-KO mice were indeed more active than their WT littermates in the empty arena (genotype effect: F_1,39_ = 15.65, *p* = 0.0003), but this phenotype was not attenuated by CBDV regardless of the dose (genotype × treatment interaction: F_2,39_ = 2.23; NS). Although there was a main effect of CBDV treatment (F_2,39_ = 11.29, *p* = 0.0001), this was explained by CBDV-induced hyperactivity at the dose of 20 mg/kg in both WT and KO mice (Figure 2a). To assess locomotor habituation, we plotted the total distance traveled in 5 min bins over the 20 min habituation session (Figure S2). This more detailed analysis yielded similar results, with an overall time-dependent reduction in locomotion across all experimental groups (5 min bin effect: F_3,117_ = 156.08, *p* < 0.0001), independent of the genotype (F_3,117_ = 0.69; NS) or treatment (F_6,117_ = 0.73; NS) (Figure S2, left and right).

We next examined the percentage of time spent in the center of the arena as an index of anxiety (Figure 2b). While similar between WT and *Fmr1*-KO mice controls injected with vehicle, this parameter was increased only in *Fmr1*-KO mice injected with CBDV at the highest dose of 100 mg/kg (genotype × treatment interaction: F_2,40_ = 3.54, *p* = 0.04; Figure 2b), suggesting the occurrence of an anxiolytic phenotype in *Fmr1*-KO mice when endocannabinoid signaling is highly stimulated.

*Sample phase.* During the sample phase, all mice explored similarly the two sample objects irrespective of their position). Although not significant, noteworthy is the tendency towards an increased exploration of the two objects by vehicle-injected *Fmr1*-KO mice (Figure 2c) compared to WT controls, consistent with their hyperactive phenotype reported in Figure 2a. While without effect in WT mice, CBDV treatment decreased the object exploration time in *Fmr1*-KO animals which resulted in an almost significant genotype × treatment interaction (F_2,40_ = 3.21, *p* = 0.051; Figure 2c).

*Test phase.* During the test phase (Figure 2d), WT control but not *Fmr1*-KO mice injected with vehicle showed a recognition index that was significantly above chance level (one sample *t*-test versus 50%: WT-VEH, t_(7)_ = 3.27; *p* = 0.01, KO-VEH, t_(7)_ = 1.21; NS). CBDV treatment at both doses did not rescue the memory deficits shown by *Fmr1*-KO and impaired object recognition in WT mice (Figure 2d). Performance of the two CBDV-treated WT and KO groups was indeed not different from chance level (one sample *t*-test versus 50% chance level: WT-20: t_(7)_ = 1.02; NS; WT-100: t_(6)_ = 0.69; NS; KO-20: t_(6)_ = −0.64; NS; KO-100: t_(7)_ = 1.91; NS; Figure 2d).

- Three-compartment test for sociability and social novelty

**Figure 3:**
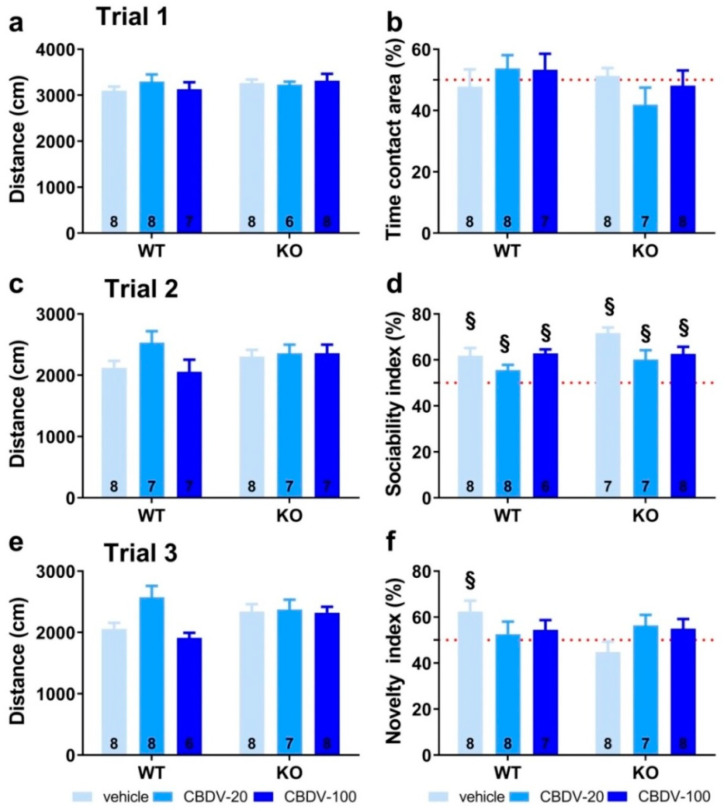
Effects of adult subchronic CBDV administration on sociability and social novelty preference in the three-compartment paradigm (Study 1). Locomotion (**a**,**c**,**e**), percent sociability (**b**,**d**) and social novelty recognition (**f**) scores are shown for each of the three 5 min trials of the test. These included a first trial of habituation to the apparatus containing the empty stimulus cages (**a**,**b**), a second trial of sociability (**c**,**d**) aimed at assessing the percent preference for a social versus non-social novel stimulus (juvenile male mouse versus inanimate object) and a third trial of social novelty preference (**e**,**f**) assessing the percent preference for a novel versus familiar stimulus mouse. Data are expressed as mean ± SEM. Numbers in histograms indicate sample size for each group. § versus chance level (50%, red dotted line).

In trial 1 (habituation phase), all vehicle- and CBDV-treated mutant and WT mice traveled the same distance in the apparatus (genotype (F_1,39_ = 0.24; NS), treatment (F_2,39_ = 0.88; NS) and genotype × treatment (F_2,39_ = 0.64; NS); Figure 3a) and equally explored the two stimulus cages (genotype × treatment interaction: F_2,40_ = 1.31; NS), indicating no bias for any of the two side compartments (Figure 3b).

In trial 2 (sociability) shown in Figure 3c, again no difference was found on locomotion (genotype (F_1,40_ = 1.41; NS), treatment (F_2,40_ = 1.57; NS) and genotype × treatment (F_2,40_ = 2.27; NS)). All groups preferentially explored the social stimulus compared to the inanimate stimulus, as demonstrated by the mean percentage time spent in the area containing the juvenile male mouse that was significantly above 50% (one sample *t*-test versus 50%: *p* < 0.05 for all groups; Figure 3d). The absence of sociability deficit in KO-VEH mice was expected, based on previous data from our group and other studies (reviewed in [36]).

In trial 3 (novelty), locomotor activity did not differ significantly between experimental groups (genotype (F_1,40_ = 0.97; NS), treatment (F_2,40_ = 2.57; NS) and genotype × treatment interaction (F_2,40_ = 1.84; NS; Figure 3e)). In contrast to trial 2, only the WT-VEH control group showed a preference for the novel social stimulus (one sample *t*-test versus 50% chance level: t_(7)_ = 2.63; *p* = 0.03; Figure 3f), indicating a deficit in *Fmr1*-KO mice that was, however, not rescued by the CBDV treatment, regardless of the dose (one sample *t*-test versus 50% chance level: KO-VEH, t_(7)_ = −1.14, NS; KO-CBDV-20, t_(6)_ = 1.39, NS; KO-CBDV-100: t_(7)_ = 1.19, NS; Figure 3f)). Preference for the novel social stimulus was abolished in WT mice treated with both doses of CBDV (20 mg/kg dose: one sample *t*-test versus 50% chance level: t_(7)_ = 0.47, NS; 100 mg/kg dose: t_(6)_ = 1.04, NS; Figure 3f).

- Direct social interaction with an adult female

**Figure 4:**
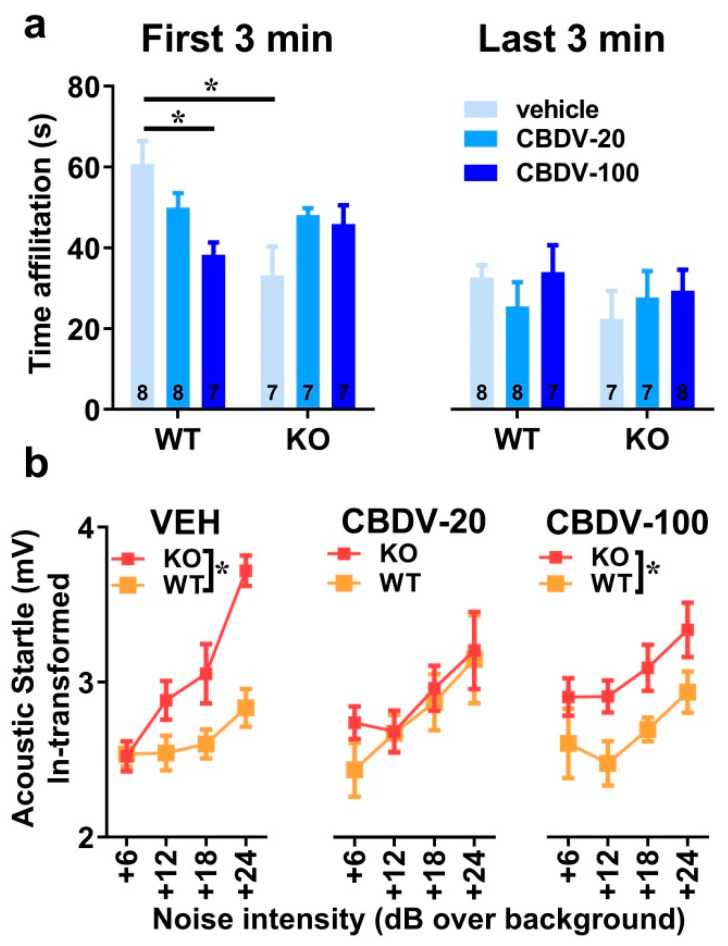
Effects of subchronic adult CBDV treatment on social interaction and acoustic startle response (Study 1). Time spent performing direct affiliative behaviors (including sniffing and contact) towards an unfamiliar adult NMRI female mouse was assessed during the first and second 3 min time bins of a 6 min interaction session (**a**). Body startle (ln-transformed to meet the normality assumptions of parametric ANOVA) was measured in response to acoustic stimuli of 6, 12, 18 and 24 dB over a background of 66 dB (**b**). Data are expressed as mean ± SEM. Numbers in histograms indicate sample size for each group. * *p* < 0.05 from post hoc comparisons following a significant interaction (**a**) or from separate ANOVAs in each treated group (**b**).

The 6 min interaction session was analyzed using two consecutive bins of 3 min in order to assess habituation to the social stimulus (Figure 4a). Most social interactions, as measured by the amount of time spent in affiliative behaviors, were displayed during the first 3 min of the interaction session and decreased afterwards independently of genotype or CBDV treatment (bin-effect: F_1,37_ = 29.91, *p* < 0.0001). When restricted to the first bin of 3 min, a significant genotype × treatment interaction (F_2,37_ = 10.16, *p* = 0.0003) emerged with *Fmr1*-KO-VEH mice exhibiting a significant decrease in the affiliation time compared to WT-VEH mice (Figure 4a, left). However, despite a trend, both doses of CBDV failed in rescuing this impaired social phenotype. In contrast, in WT mice, the CBDV treatment at the dose of 100 mg/kg decreased the amount of affiliative behavior (Figure 4a, left). During the last bin of 3 min, no between group differences were observed, all comparisons being non-significant (Figure 4a right).

- Acoustic startle response

As expected, body startle response shown in Figure 4b increased with noise intensity in all groups (intensity effect: F_3,120_ = 21.13, *p* < 0.0001). *Fmr1*-KO mice injected with vehicle showed an overall startle hyper-responsiveness compared to WT-vehicle controls which was attenuated only in *Fmr1*-KO mice treated with the 20 mg/kg dose of CBDV (Figure 4b), as demonstrated by separate ANOVAs yielding genotype effects in VEH (F_1,14_ = 10.84, *p* = 0.005) and CBDV-100 (F_1,13_ = 8.29, *p* = 0.01) groups, but not in the CBDV-20 group (F_1,13_ = 0.60; NS).

#### 3.1.2. Brain Analyses

- Hippocampus

The effects of CBDV administration on the expression patterns of inflammatory (*TNF-α*, *IL1b*, *IL-6*, *IL-10*, *CD45* or *CD11b*) and plasticity (*BDNF*) markers were analyzed in the CA1, CA3 and dentate gyrus of hippocampus of WT and *Fmr1*-KO mice (Table 1). Neither the *Fmr1* mutation nor CBDV significantly affected RNA levels of these genes in the CA1 area (Table 1). In contrast, in the CA3 area, RNA expression of the pro-inflammatory cytokine gene *TNF-α* was reduced in *Fmr1*-KO mice compared to WT mice (genotype effect: F_1,38_ = 7.76, *p* = 0.008; Table 1). However, despite a trend, this effect was not rescued by the CBDV treatment, regardless of the dose (genotype × treatment interaction: F_2,38_ = 2.55; NS).

Despite the lack of genotype effect (F_2,39_ = 0.39; NS), a significant effect of CBDV treatment was observed on the pro-inflammatory cytokine gene *IL-1b* (F_2,39_ = 5.13, *p* = 0.01; Table 1) whose RNA expression was increased in the CA3 of WT, but not of *Fmr1*-KO, mice injected at the dose of 100 mg/kg compared to vehicle-injected WT mice (genotype × treatment effect close to reaching significance: F_2,39_ = 2.99, *p* = 0.06; treatment effect from separate ANOVAs in WTs: F_2,19_ = 6.87, *p* = 0.006, in KOs: F_2,20_ = 0.17, NS, Table 1). CBDV also affected the *CD-45* gene involved in cytokine production and proliferation of T cells (treatment effect: F_2,39_ = 7.0, *p* = 0.002; Table 1). RNA expression of *CD-45* increased in a dose-dependent manner, an effect that seemed more marked in *Fmr1*-KO mice. However, there was no significant genotype effect (F_1,39_ = 1.49; NS) or genotype × treatment interaction (F_2,39_ = 2.5, NS). There were no significant between-group changes in the RNA levels of the neurotrophic factor *BDNF*, the anti-inflammatory cytokines *IL-6* and *IL-10* or the microglia marker *CD11b* (Table 1).

In the dentate gyrus, RNA levels of BDNF were increased in *Fmr1*-KO mice compared to WT animals injected with vehicle (genotype × treatment interaction: F_3,40_ = 3.27; *p* = 0.048; Table 1), but they were not significantly affected by CBDV treatment (F_2,40_ = 0.30, NS). IL-1b RNA levels were overall increased in *Fmr1*-KO mice compared to their WT littermates (genotype effect: F_1,38_ = 4.75, *p* = 0.036, Table 1), without any significant effect of CBDV treatment (genotype × treatment: F_2,38_ = 3.05, NS). As to the other neuroinflammatory markers, neither the *Fmr1* mutation nor the CBDV treatment affected IL10, IL6, CD11b, CD45 or TNFα RNA levels (Table 1).

- Prefrontal cortex

In the prefrontal cortex, none of the brain markers examined were significantly affected by either the Fmr1 mutation or the CBDV treatments (Table 1).

### 3.2. Study 2: Effects of Chronic Administration of CBDV at Weaning

The body weight gain occurring between 3 and 8 weeks of age, i.e., before and after the 5 weeks of CBDV treatment, did not differ among experimental groups (genotype, treatment effects and their interaction: F_1,41_ = 0.23, F_2,41_ = 1.27, F_2,41_ = 0.16, NS). The mean ± SEM values of the percent body weight gain for each group were the following: 105.71 ± 20.01% (WT-VEH), 86.03 ± 10.10% (WT-CBDV-20), 79.88 ± 13.02% (WT-CBDV-100), 80.24 ± 15.69% (KO-VEH), 95.50 ± 10.02% (KO-CBDV-20) and 82.28 ± 10.22% (KO-CBDV-100).

#### 3.2.1. Behavioral Profiling

- Elevated plus maze

The effects of juvenile chronic administration of CBDV on anxiety-like behavior in the elevated plus maze are presented in Figure S3. Anxiety levels, assessed by the percent time spent in the open arms, were reduced in *Fmr1*-KO mice independently of CBDV treatment (genotype effect: F_1,39_ = 13.09, *p* = 0.0008; genotype × treatment interaction: F_2,39_ = 1.75, NS; Figure S3a). Locomotor activity, indexed by the number of total arm entries, was overall enhanced in *Fmr1*-KO mice compared to their WT littermates, and this hyperactive phenotype was unaffected by CBDV treatment (genotype effect: F_1,39_ = 14.97, *p* = 0.0004; genotype × treatment interaction: F_2,39_ = 1.55, NS; Figure S3b).

- Object recognition test

**Figure 5:**
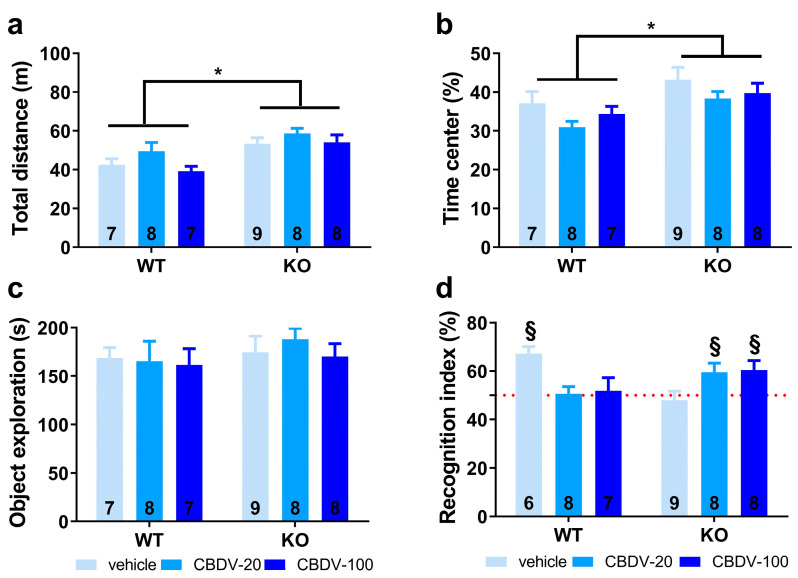
Effects of juvenile chronic CBDV administration on object recognition memory (Study 2). Locomotor activity (**a**) and anxiety-like behavior (**b**) were measured during the 20 min habituation session in the empty open field. The time spent exploring both objects (**c**) and the novel object recognition index (**d**) were computed during the 5 min sample and test phases, respectively. All behavioral parameters were measured in WT and *Fmr1*-KO mice treated with vehicle solution, CBDV at the dose of 20 mg/kg (CBDV-20) or 100 mg/kg (CBDV-100). Data are expressed as mean ± SEM. Numbers in histograms indicate sample size for each group. * *p* < 0.05; § versus chance level (50%, red dotted line).

Habituation phase. As expected, *Fmr1*-KO mice were more active than their WT littermates (genotype effect: F_1,41_ = 17.65, *p* = 0.0001; Figure 5a). However, CBDV treatment, regardless of the dose, did not normalize this locomotor phenotype (genotype × treatment interaction: F_2,41_ = 0.37; NS). All experimental groups displayed locomotor habituation as shown by a time-dependent reduction in locomotion (5 min-bin effect: F_3,123_ = 117.44, *p* < 0.0001; Figure S4) independent of the genotype (genotype × 5 min-bin interaction: F_3,123_ = 1.64; NS) or treatment (treatment × 5 min-bin interaction: F_3,123_ = 1.68; NS).

Anxiety levels appeared slightly reduced in *Fmr1*-KO mice compared to their WT littermates as shown by the increased time spent in the center of the arena (genotype effect: F_1,41_ = 9.42, *p* = 0.004; Figure 5b). However, this anxiolytic phenotype in the open field was not modified by CBDV treatments (genotype × treatment interaction: F_2,41_ = 0.09; NS).

Sample phase. All mice explored similarly the two sample objects irrespective of their position, indicating the absence of any spatial bias There was no significant difference in object exploration across experimental groups as shown by the absence of genotype or CBDV treatment effects (F_1,41_ = 0.56, NS and F_2,41_ = 0.24, NS) or a genotype × treatment interaction (F_2,41_ = 0.03; NS; Figure 5c).

Test phase. During the test phase, as already observed in Study 1, a clear object recognition deficit was detected in *Fmr1*-KO-VEH (one sample *t*-test versus 50%: t_(8)_ = −0.53, NS) but not in WT-VEH mice that preferentially explored the novel object (t_(5)_ = 6.04, *p* = 0.002; Figure 5d). This deficit was eliminated by both doses of CBDV (one sample *t*-test versus 50%: *Fmr1*-KO CBDV-20, t_(7)_ = 2.59, *p* = 0.04; *Fmr1*-KO-CBDV-100, t_(7)_ = 2.62, *p* = 0.03; Figure 5d). In contrast, CBDV treatments impaired recognition memory of WT mice that was not significantly different from chance level (one sample *t*-test versus 50%: t_(7)_ = 0.22, *NS* for *Fmr1*-KO-CBDV-20 mice and t_(6)_ = 0.36, *NS* for *Fmr1*-KO-CBDV-100 mice; Figure 5d).

- Three-compartment test for sociability and social novelty

**Figure 6:**
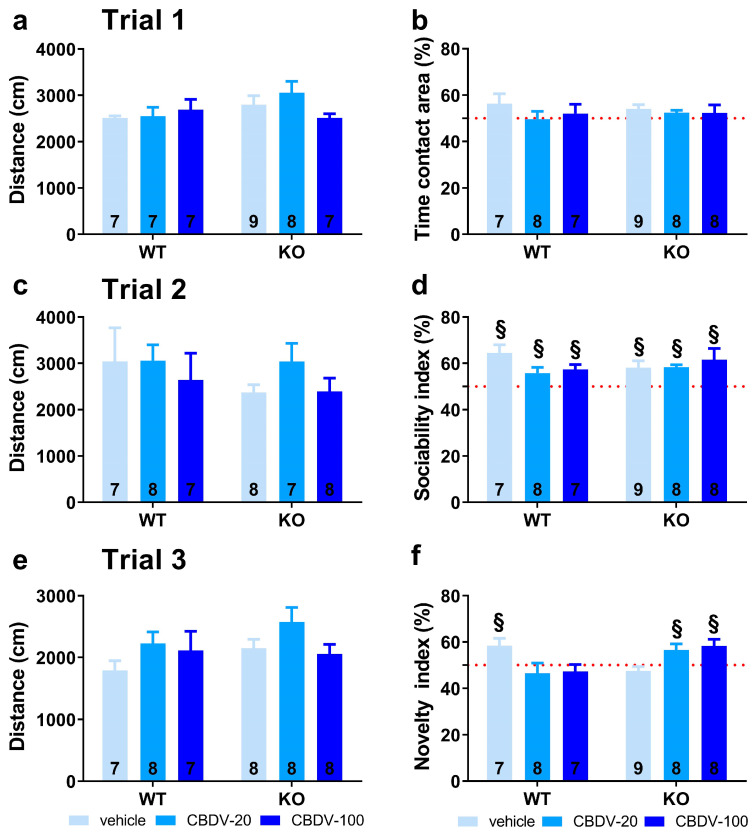
Effects of juvenile chronic CBDV administration on sociability and social novelty preference in the three-compartment paradigm (Study 2). Locomotion (**a**,**c**,**e**), percent sociability (**b**,**d**) and social novelty recognition (**f**) scores are shown for each of the three 5 min trials of the test. These included a first trial of habituation to the apparatus containing the empty stimulus cages (**a**,**b**), a second trial of sociability (**c**,**d**) aimed at assessing the percent preference for a social versus non-social novel stimulus (juvenile male mouse versus inanimate object) and a third trial of social novelty preference (**e**,**f**) assessing the percent preference for a novel versus familiar stimulus mouse. Data are expressed as mean ± SEM. Numbers in histograms indicate sample size for each group. § versus chance level (50%, red dotted line).

In trial 1 (habituation phase), no main effects of genotype (F_1,41_ = 0.83, NS), treatment (F_2,41_ = 0.86, NS) or genotype × treatment interaction (F_2,41_ = 0.09, NS) were found on locomotor activity (Figure 6a). All vehicle- and CBDV-treated mice explored similarly the two side compartments (one sample t-test versus 50%: NS for all groups; Figure 6b), indicating no particular spatial bias.

In trial 2 (sociability), no difference in the distance traveled across groups was observed (genotype and treatment effects: F_1,41_ = 0.08, F_2,41_ = 1.45, NS; genotype × treatment interaction: F_2,41_ = 0.62, NS; Figure 6c). All mice preferred to explore the social versus the inanimate stimulus, as shown by the mean sociability index that was significantly above 50% in all experimental groups (one sample t-test versus 50%: *p* < 0.05 for all groups; Figure 6d).

In trial 3 (novelty), locomotor activity did not differ between experimental groups (genotype and treatment effects: F_1,40_ = 1.71, NS; F_2,40_ = 2.52, NS; genotype × treatment interaction: F_2,40_ = 0.69, NS; Figure 6e). As opposed to WT-VEH control mice which showed a preference for the novel social stimulus (one sample *t*-test versus 50%: t_(5)_ = 4.17, *p* = 0.01), the *Fmr1*-KO-VEH group exhibited a clear and expected lack of preference for social novelty (t_(8)_ = −1.31; NS). This deficit was prevented in mutant mice by CBDV chronic administration at both 20 mg/kg and 100 mg/kg doses (one sample *t*-test versus 50% in *Fmr1*-KO-CBDV-20 mice: t_(7)_ = 2.50; *p* = 0.04; in *Fmr1*-KO-CBDV-100 mice: t_(7)_ = 2.88; *p* = 0.02; Figure 6f). In contrast, the CBDV treatment was deleterious in WT mice which failed to exhibit a significant preference for the novel social stimulus at both 20 mg/kg and 100 mg/kg doses. The performance of WT mice treated with CBDV was not different from chance level (one sample *t*-test versus 50%: WT-CBDV-20 group, t_(7)_ = −0.79, NS; WT-CBDV-100 group: t_(6)_ = −0.93, NS; Figure 6f).

- Direct social interaction with an adult female

**Figure 7:**
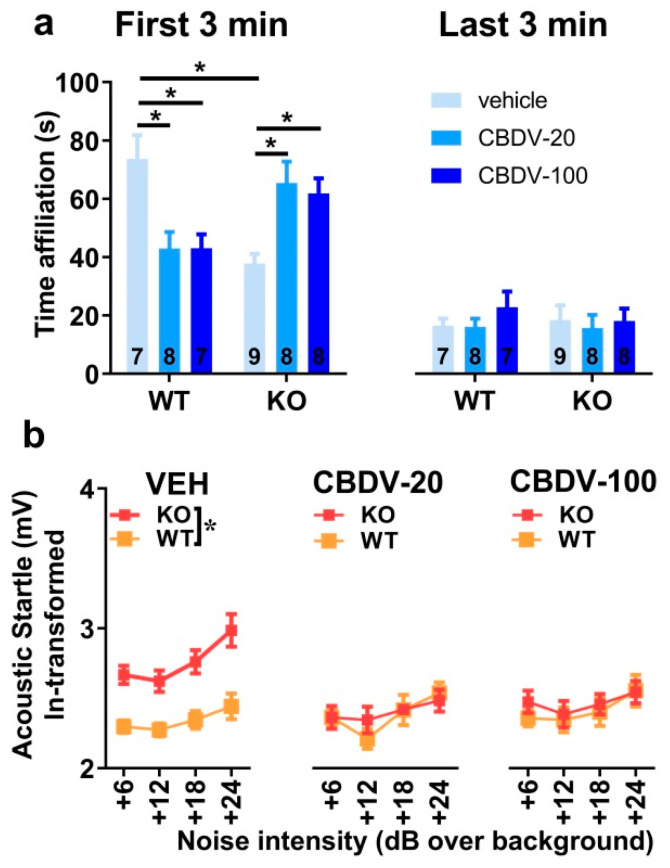
Effects of juvenile chronic CBDV treatment on social interaction and acoustic startle response (Study 2). Time spent performing direct affiliative behaviors (including sniffing and contact) towards an unfamiliar adult NMRI female mouse was assessed during the first and second 3 min time bins of a 6 min interaction session (**a**). Body startle (ln-transformed to meet the normality assumptions of parametric ANOVA) was measured in response to acoustic stimuli of 6, 12, 18 and 24 dB over a background of 66 dB (**b**). Data are expressed as mean ± SEM. Numbers in histograms indicate sample size for each group. * *p* < 0.05 from post hoc comparisons following a significant interaction (**a**) or from separate ANOVAs in each treated group (**b**).

Social habituation occurred over a 6 min interaction session that was separated into two time bins of 3 min each. There was a main effect of time bin (F_1,41_ = 225.45, *p* < 0.0001; Figure 7a), confirming that the highest levels of social affiliative behaviors are displayed during the first 3 min of interaction (Figure 7a, left). As for study 1, these behaviors decreased during the second time bin independently of genotype or CBDV treatment (Figure 7a, right). The ANOVA revealed group differences on social interaction only during the first time bin (interaction genotype × treatment × 3 min bin: F_2,41_ = 16.12, *p* < 0.0001) with *Fmr1*-KO-VEH mice displaying a significant reduction of their affiliation time compared to WT-VEH mice (Figure 7a, left). This impairment during the first 3 min was attenuated following CBDV treatments at both doses. However, CBDV was not beneficial for WT mice which exhibited a reduced affiliation time for both low and high doses of CBDV (genotype × treatment interaction on the first 3 min-bin: F_2,41_ = 15.95, *p* < 0.0001; Figure 7a, left). During the last bin of 3 min, no between-group differences were observed, all comparisons being non-significant (Figure 7a, right).

- Acoustic startle response

All groups exhibited a body startle response which increased with stimulus intensity (intensity effect: F_3,123_ = 11.65, *p* < 0.0001; Figure 7b). *Fmr1*-KO mice showed an overall startle hyper-responsiveness (genotype effect: F_1,41_ = 12.41, *p* = 0.001). This phenotype was abolished by CBDV administration (genotype × treatment interaction: F_2,41_ = 7.68, *p* = 0.002; Figure 7b), with both the 20 mg/kg and 100 mg/kg doses being equally efficacious. CBDV treatments did not affect body startle response in WT-mice, indicating no side effects.

#### 3.2.2. Brain Analyses

- *Hippocampus*

Expression patterns of inflammatory (*TNF-α*, *IL1b*, *IL-6*, *IL-10*, *CD45* or *CD11b*) and plasticity (*BDNF*) markers were marginally affected in the CA1 area of the hippocampus of WT and *Fmr1*-KO mice (Table 2). The only statistically significant difference concerned RNA expression of the *IL-6* gene (interaction genotype × treatment: F_2,36_ = 2.27, *p* = 0.049; Table 2). However, all post hoc comparisons failed to reach significance. In the CA3 area, amongst all brain markers analyzed (Table 2), the only significant differences were observed for the pro-inflammatory cytokine gene *CD11b* (Table 2). Its RNA expression was not altered in *Fmr1*-KO compared to WT mice (genotype: F_1,41_ = 0.22, NS). However, CBDV increased *CD11b* expression in both WT and KO mice (treatment effect: F_2,41_ = 15.23, *p* < 0.0001; Table 2), the dose of 20 mg/kg being the most effective.

In the dentate gyrus, RNA levels of the *CD45* gene were increased only by the 100 mg/kg dose of CBDV in mice of both genotypes (treatment effect: F_2,40_ = 5.13, *p* = 0.01; Table 2). Likewise, the highest dose of CBDV increased *CD11b* RNA expression in all mice (treatment effect: F_2,40_ = 3.84, *p* = 0.03; Table 2). There was, however, no genotype effect for these two genes (Table 2). For the *IL-10* gene, there was a genotype × treatment interaction (F_2,37_ = 3.67, *p* = 0.03; Table 2), but all post hoc comparisons failed to reach statistical significance. Neither the *Fmr1* mutation nor CBDV significantly affected RNA levels of all the other brain markers (Table 2).

- *Prefrontal cortex*

RNA levels of *BDNF* were similar in *Fmr1*-KO and WT mice (genotype effect: F_1,38_ = 0.10, NS). CBDV altered RNA expression of the *BDNF* gene in both WT and *Fmr1*-KO mice in a dose-dependent manner (Table 2). While increased by the 20 mg/kg dose, RNA levels of *BDNF* were decreased by the 100 mg/kg dose (treatment effect: F_2,38_ = 3.32, *p* = 0.047). Neither the *Fmr1* mutation nor CBDV significantly affected RNA levels of all the other brain markers (Table 2).

## 4. Discussion

Our findings provide for the first time a full characterization of the neurobehavioral effects of CBDV administration in the *Fmr1*-KO mouse model of FXS. In the first study (Study 1) which consisted of administering CBDV for 10 days starting at adult age (3 months), we show a rescue effect of CBDV at the 20 mg/kg dose only on the startle hyper-responsiveness of *Fmr1*-KO mice and, at both doses, on their altered BDNF levels in the dentate gyrus. In the second study (Study 2), when treatments started at weaning (PND 21) and were given chronically for 5 weeks, we instead demonstrated a larger array of beneficial effects of CBDV in *Fmr1*-KO mice. All FXS-like behavioral phenotypes in these mutants were prevented, except hyperactivity. Furthermore, in Study 2, although marginal, CBDV modified the expression of several inflammatory brain markers in mice of both genotypes. Overall, our results indicate the emergence of robust genotype and main treatment effects, as well as interactions, for selected behavioral measures. These effects are summarized in Table 3.

Concerning the main behavioral effects of genotype (Table 3), we confirmed in both studies the most robust behavioral phenotypes of the *Fmr1*-KO mouse model, as previously reported by us and others (for a review, see [53]). These included hyperactivity in the open field [39,40,54,55,56,57,58,59,60,61,62,63,64,65,66,67], reduced direct social interaction [36,39,41,55,64,68], lack of preference for social novelty [38,41,54,55,59,64,69,70], sensory hyper-responsiveness in the acoustic startle paradigm [42,71] and deficits in novel object memory [40,54,55,72]. We also found anxiety-like behavior to be an inconsistent phenotype of this mouse model. Indeed, whereas we did not observe any genotype difference in Study 1, in accordance with previous studies by us [38] and others (e.g., [57,60,64,73,74]), reduced anxiety-like behaviors of *Fmr1*-KO mice were detected in the elevated plus maze and open field test in Study 2, as previously reported as well [10,58,64,65,75]. Our data therefore further underline the inconsistency of the emotional phenotype of *Fmr1*-KO mice [53], in contrast with what is typically observed in FXS patients who exhibit a robust increase in anxiety levels. As for sociability (in trial 2 of the three-compartment test), the lack of a *Fmr1*-KO phenotype was confirmed in both studies, as previously demonstrated by us [38,41] and others [54,55,59,64,69,70]. It should be noted that the hyperactivity exhibited by *Fmr1*-KO mice in the open field of both Study 1 and Study 2 was less marked than what was previously reported in the literature, including by us (for a review see [36]), as this parameter emerged only as an overall genotype difference but failed to reach significance when WT-VEH and *Fmr1*-KO-VEH mice were compared. This finding may be explained, at least in part, by the confounding stressful effects of the repeated intraperitoneal injections performed in both studies.

The behavioral phenotypes of *Fmr1*-KO mice were normalized by CBDV depending on the treatment schedule (Table 3). While the efficacy of subchronic CBDV treatment at adulthood was limited to acoustic startle and at the lowest dose (Study 1), chronic administration of both doses of CBDV from weaning (Study 2) eliminated all the behavioral alterations of *Fmr1*-KO mice, except hyperactivity and reduced anxiety, thus preventing the most relevant symptoms associated with the FXS. These results therefore confirm our hypothesis that juvenile chronic CBDV administration is more efficacious than a subchronic treatment delivered at the adult stage. They further corroborate the view that early timing of intervention is critical for alleviating the behavioral alterations induced by the *Fmr1* mutation [39,40] and identify the juvenile/adolescence phase as a sensitive period for pharmacological targeting in preclinical models of NDDs. Although such a differential effect could be anticipated based on the high levels of neurobehavioral plasticity characterizing the adolescent phase in mice as in humans [76,77], it does not represent an obvious finding. Indeed, previous studies have described the efficacy of 10-day pharmacological treatments other than phytocannabinoids (e.g., metformin and potassium-channel agonists [78,79]) in reversing multiple neurobehavioral phenotypes of *Fmr1*-KO mice when administered at adulthood. Interestingly, these treatments were also able to rescue the alterations in hippocampal spine density and morphology shown by *Fmr1*-KO mice at adulthood, a brain phenotype that we did not assess here and which may represent a key mechanism for the full rescue of *Fmr1*-KO behavioral phenotypes, as previously suggested [39,78,79].

While CBDV had marginal effects in terms of the behavioral rescue of mutant mice when administered subchronically at adulthood (Study 1), it also exerted several behavioral effects in WT mice (Table 3). Interestingly, these effects were highly comparable to those observed in Study 2 (Table 3), thus suggesting that the timing of delivery does not represent a critical determinant for the behavioral effects of CBDV in WT animals, in contrast to its effects in *Fmr1* mutants. In both studies, cognitive and social deficits occurred in WT mice treated with both doses of CBDV in the novel object recognition and 3-compartment tests, as well as in the direct social interaction paradigm, while anxiety was unaltered (Table 3). It can be hypothesized that CBDV may induce beneficial effects in *Fmr1*-KO mice by normalizing the ECS hyper-functionality of these animals, while opposite detrimental effects are induced when a “normally” functioning ECS is modulated, as in the case of WT subjects. This hypothesis could be tested by exploring the differential effects of CBDV in our *Fmr1*-KO and WT mice on ECS functionality, e.g., by assessing mTOR activation, since this signaling pathway is altered in *Fmr1*-KO mice [9]. To the best of our knowledge, this is the first study reporting clear detrimental behavioral outcomes of phytocannabinoids in WT mice, as no or minor effects were described in previous preclinical studies that have examined the consequences of CBD [80] and CBDV [6] treatments in other mouse models of NDDs. Close inspection of the findings obtained using the mouse model of Rett syndrome indicates that CBDV at doses of 20 and 100 mg/kg induced a lack of social preference (close to chance level) in the three-compartment test (Figure 2d in [6]). Differences in the genetic background (as only our mice are on a pure B6) could contribute to explaining the discrepancies with previous studies. Further experiments using WT mice bred from WT breeders would be necessary to generalize our results to the B6 strain, as our WT controls here were bred from *Fmr1*-HET females and could perhaps be oversensitive to the effects of CBDV. Previous studies have indeed shown that WT mice of the *Fmr1* line present several behavioral differences compared to WT animals of the same background obtained from WT breeders [81].

In contrast to the behavioral phenotypes, the brain alterations displayed by *Fmr1*-KO mice injected with vehicle were less robust and highly variable between our two studies (Table 1 and Table 2). In Study 1, *Fmr1*-KO animals displayed reduced expression of *TNF-α* in the CA3 and higher levels of *IL1b* and *BDNF* in the DG, while no difference emerged in Study 2. The discrepancy between the two studies may be due to differences in experimental conditions, such as the chronic versus subchronic injection regimen, and support the weakness and high variability of the brain inflammatory phenotype of the *Fmr1*-KO model. Previous results indeed suggested a subtle imbalance in hippocampal and cortical inflammatory markers in these mutants, with a decrease in some specific regions and an increase in others [40]. Similarly, the increase in *BDNF* expression restricted to the DG of *Fmr1* mutants in Study 1 was in disagreement with previous findings showing reduced *BDNF* levels in both CA1 and PFC in untreated *Fmr1*-KO mice [40]. Given the altered sensitivity to stress of Fmr1-KO mice [82,83,84,85,86] and since stress is a well-known modulator of *BDNF* expression, especially in the hippocampus [87,88,89,90,91,92], increased stress levels due to repeated intraperitoneal injections in Study 1 may explain this discrepancy, in contrast to Study 2 wherein the longer injection regimen may have resulted in a less stressful experience because of potential habituation effects. Alternatively, the enhanced *BDNF* levels found in *Fmr1*-KO mice could reflect an imbalance in other neurotrophic factors. For instance, previous studies have demonstrated a reduction in the hippocampal expression of the nerve growth factor associated with a tendency to increased hippocampal *BDNF* levels in another mouse model of developmental disorder, the Rett syndrome [6]. Independent of the selected interpretative hypothesis, our results on BDNF support a region-specific role of this neurotrophin since changes were detected in the dentate gyrus and not in other hippocampal subregions. This observation is in line with previous studies demonstrating changes in *BDNF* expression specifically in the dentate gyrus following various manipulations such as caloric restriction [93], stress exposure [94] or treatments with antidepressants [95] or intoxicants [96] which alter neurogenesis in the DG, thus highlighting a possible connection between BDNF and neurogenesis.

CBDV was able to affect several brain markers in both WT and *Fmr1*-KO mice, although these effects were limited by the high variability within and between brain regions (Table 1 and Table 2). These marginal pharmacological effects were detected in both studies and appeared slightly more pronounced in Study 2, where they emerged across more brain areas and affected the RNA expression of both inflammatory and plasticity markers. Hence, these findings corroborate the higher efficacy of CBDV in preventing behavioral impairments when delivered chronically during the juvenile period. Although region- and dose-specific differences exist between our two studies, the effects of CBDV seemed to consistently increase the expression of *CD11b* and *CD45* (Table 1 and Table 2) in both WT and *Fmr1*-KO mice. This observation is in accordance with the effects of CBDV in WT controls and *Fmr1*-KO mice exposed to dietary supplementation of omega-3 fatty acids, a non-pharmacological treatment that translated into beneficial behavioral effects [40]. However, it contrasts with findings obtained in the valproic acid (VPA) rat model of ASD [35] in which activated microglia in the hippocampus was rescued by CBDV. This apparent discrepancy may be due to differences in the basal inflammatory state existing between Fmr1-KO mice (no effect of the mutation on *CD11b* and *CD45* expression) and VPA-treated rats (increased *CD11b* and *CD45* expression). It is possible that the basal level of inflammation could influence the outcome of a given pharmacological treatment. In a condition wherein cytokines and microglia activity remain within the physiological range to ensure normal synaptic plasticity (for review see [97]), a treatment-induced increase in neuroinflammation may remain tolerable without compromising therapeutic effects whereas in an existing inflammatory condition with cytokine overproduction or microglia activation, the same treatment may appear deleterious and induce neurobehavioral impairments (for review see [98]). Furthermore, it is important to underline that our study examined mRNA expression levels which correlate only partially with functional protein levels due to post-transcriptional regulation of gene expression. Caution is therefore required when relating changes in brain marker expression to behavioral outcomes. This may explain, at least in part, some discrepancies between studies on the effects of endocannabinoids on cerebral expression levels of neuroinflammatory and plasticity markers.

The marginal between-group differences emerging on the brain markers that we evaluated in the present study suggest that other complementary mechanisms may underlie the behavioral effects of CBDV. Like its phytocannabinoid analog CBDl, CBDV is thought to exhibit neuroprotective anti-inflammatory properties [28] that may act in concert with the modulation of ECS functionality to support the beneficial effects of CBDV, especially its greater efficacy during the early stage of the FXS. Loss of FMRP in *Fmr1* mutants is thought to disrupt ECS signaling [9,10,11,12], leading to neuronal hyper-excitability and the FXS- and ASD-like behavioral phenotypes of *Fmr1*-KO mice. It is therefore likely that CBDV may rescue the altered endocannabinoid tone in the brain of *Fmr1* mutants, for instance by normalizing the endocannabinoid-degrading enzymes, fatty acid amide hydrolase (FAAH) and monoacylglycerol lipase (MAGL), as already shown in the valproic acid rat model of ASD [35].

## 5. Conclusions

Overall, these data demonstrate that CBDV, when administered chronically and starting at juvenile age, holds a solid therapeutic potential for FXS as it prevented the most relevant behavioral alterations shown by *Fmr1*-KO mice. Early timing and chronic duration of treatment appear as critical determinants to ensure the beneficial effects of CBDV. The efficacy of CBDV is comparable to that of other pharmacological treatments previously tested in the *Fmr1*-KO mouse model. For instance, valuable neurobehavioral effects were reported following chronic administration of the nonspecific GABA receptor agonist acamprosate [99], the antibiotic minocycline [100,101] or the selective mGlu5 inhibitor CTEP [71]. While the administration of acamprosate was performed at adulthood [99], the other treatments were administrated earlier, either at adolescence [71,100] or starting from birth [101], thus supporting our observation of a higher sensitivity of earlier developmental phases to the effects of pharmacological interventions. Interestingly, despite their different pharmacological targets (GABAergic [99] and glutamatergic receptors [71] or matrix metalloproteinases (MMP) [100,101]), these treatments were shown to normalize the brain excitatory/inhibitory (E/I) imbalance reported in *Fmr1*-KO mice by rescuing overreactive extracellular signal-regulated kinase 1 and 2 (ERK1/2) and mTOR signaling [71,99]. The same pathways were also normalized by subchronic adult administration of the MMP inhibitor metformin [78] in *Fmr1*-KO mice and are known to be modulated by phytocannabinoids [102]. Furthermore, E/I imbalance has been suggested as a key common etiopathological mechanism involved in several neurodevelopmental disorders [103]. These results thus encourage future clinical studies using phytocannabinoids for treating not only FXS but also other neurodevelopmental disorders.

## Figures and Tables

**Table 1:** Effects of subchronic adult CBDV administration on the RNA expression of plasticity and inflammatory brain markers (Study 1).

Brain Region	Brain Marker	SUBCHRONIC TREATMENT AT ADULTHOOD						
		VEH		CBDV-20		CBDV-100		Significant Differences
		WT	KO	WT	KO	WT	KO	
*CA1*	*BDNF*	1.00 ± 0.03	1.01 ± 0.02	0.99 ± 0.05	0.98 ± 0.03	0.93 ± 0.03	0.96 ± 0.04	
	*IL-10*	1.18 ± 0.23	1.11 ± 0.21	1.57 ± 0.44	1.16 ± 0.24	0.65 ± 0.12	0.82 ± 0.15	
	*IL-1β*	1.03 ± 0.10	0.95 ± 0.10	0.86 ± 0.07	1.09 ± 0.12	1.00 ± 0.07	1.14 ± 0.21	
	*IL-6*	1.02 ± 0.08	1.05 ± 0.13	1.33 ± 0.22	1.10 ± 0.24	0.93 ± 0.13	0.95 ± 0.10	
	*CD11b*	1.00 ± 0.02	1.00 ± 0.02	1.12 ± 0.04	0.95 ± 0.04	1.05 ± 0.04	1.04 ± 0.07	
	*CD45*	1.00 ± 0.03	0.95 ± 0.05	0.92 ± 0.05	0.88 ± 0.03	0.95 ± 0.06	1.00 ± 0.07	
	*TNF-α*	1.11 ± 0.18	0.91 ± 0.21	1.12 ± 0.15	0.84 ± 0.12	1.13 ± 0.20	1.04 ± 0.20	
*CA3*	*BDNF*	1.01 ± 0.04	1.10 ± 0.04	1.06 ± 0.06	1.04 ± 0.04	1.06 ± 0.06	1.03 ± 0.04	
	*IL-10*	1.13 ± 0.20	0.92 ± 0.23	1.07 ± 0.22	0.82 ± 0.16	1.58 ± 0.67	0.96 ± 0.14	
	*IL-1β*	1.14 ± 0.19	1.28 ± 0.25	0.94 ± 0.14	1.28 ± 0.25	2.37 ± 0.46	1.46 ± 0.26	Treatment effect (CBDV-100 > CBDV-20 and VEH) only in WT
	*IL-6*	1.06 ± 0.12	0.59 ± 0.08	1.07 ± 0.18	1.19 ± 0.18	0.91 ± 0.22	0.90 ± 0.10	
	*CD11b*	1.01 ± 0.06	0.92 ± 0.05	1.05 ± 0.10	0.97 ± 0.04	0.89 ± 0.04	0.99 ± 0.06	
	*CD45*	1.03 ± 0.09	0.92 ± 0.07	0.87 ± 0.06	1.07 ± 0.07	1.14 ± 0.08	1.26 ± 0.06	Overall treatment effect (CBDV-100 > CBDV-20 and VEH)
	*TNF-α*	1.06 ± 0.13	0.41 ± 0.04	1.03 ± 0.26	0.69 ± 0.16	0.86 ± 0.19	0.76 ± 0.09	Overall genotype effect (KO < WT)
*DG*	*BDNF*	1.00 ± 0.03	1.24 ± 0.08	1.15 ± 0.06	1.11 ± 0.06	1.14 ± 0.04	1.18 ± 0.02	Genotype effect (KO > WT) only in VEH
	*IL-10*	1.10 ± 0.18	1.05 ± 0.19	1.03 ± 0.30	1.00 ± 0.19	0.82 ± 0.15	0.74 ± 0.14	
	*IL-1β*	1.03 ± 0.10	1.22 ± 0.22	1.53 ± 0.23	1.01 ± 0.06	1.45 ± 0.35	0.72 ± 0.06	
	*IL-6*	1.17 ± 0.24	1.19 ± 0.12	1.30 ± 0.28	1.16 ± 0.13	1.14 ± 0.16	0.94 ± 0.08	
	*CD11b*	1.01 ± 0.06	1.04 ± 0.02	1.10 ± 0.05	1.02 ± 0.04	1.23 ± 0.10	1.06 ± 0.05	
	*CD45*	1.02 ± 0.08	1.19 ± 0.09	1.14 ± 0.06	0.93 ± 0.09	1.23 ± 0.14	1.08 ± 0.07	
	*TNF-α*	1.06 ± 0.13	1.04 ± 0.19	0.92 ± 0.19	0.55 ± 0.08	0.72 ± 0.11	0.78 ± 0.21	
*PFC*	*BDNF*	1.01 ± 0.04	0.95 ± 0.07	0.94 ± 0.10	0.91 ± 0.09	0.95 ± 0.06	0.92 ± 0.06	
	*IL-10*	0.93 ± 0.25	0.57 ± 0.04	1.44 ± 0.37	0.92 ± 0.18	0.64 ± 0.09	1.44 ± 0.39	
	*IL-1β*	1.05 ± 0.09	0.96 ± 0.13	1.20 ± 0.16	0.98 ± 0.09	0.86 ± 0.11	0.99 ± 0.08	
	*IL-6*	0.83 ± 0.11	0.95 ± 0.20	1.33 ± 0.24	1.06 ± 0.11	0.90 ± 0.09	1.14 ± 0.14	
	*CD11b*	1.01 ± 0.06	0.98 ± 0.11	0.97 ± 0.06	0.91 ± 0.05	0.93 ± 0.07	1.03 ± 0.04	
	*CD45*	0.55 ± 0.21	1.06 ± 0.11	0.95 ± 0.15	0.80 ± 0.03	1.05 ± 0.07	1.01 ± 0.08	
	*TNF-α*	0.56 ± 0.21	0.54 ± 0.13	0.68 ± 0.23	0.60 ± 0.12	0.59 ± 0.05	0.90 ± 0.16	

mRNA quantification was performed by RT-qPCR analysis. The relative level of expression was calculated with the comparative (2−ΔΔCT) method [51]. Data refer to bold change and are expressed as mean ± SEM. Sample size for each group was 7–9. Statistically significant differences are highlighted in yellow. CA1 = hippocampal CA1 subfield; CA3 = hippocampal CA3 subfield; DG = dentate gyrus; PFC = prefrontal cortex.

**Table 2:** Effects of chronic juvenile CBDV administration on the RNA expression of plasticity and inflammatory brain markers (Study 2).

Brain Region	Brain Marker	CHRONIC TREATMENT AT WEANING						
		VEH		CBDV-20		CBDV-100		Significant Differences
		WT	KO	WT	KO	WT	KO	
*CA1*	*BDNF*	1.00 ± 0.04	1.02 ± 0.03	1.13 ± 0.06	1.15 ± 0.06	0.90 ± 0.05	1.12 ± 0.09	
	*IL-10*	1.96 ± 0.77	1.28 ± 0.52	0.55 ± 0.25	2.54 ± 1.08	2.36 ± 1.22	0.78 ± 0.41	
	*IL-1β*	1.43 ± 0.40	1.11 ± 0.42	0.59 ± 0.19	1.73 ± 0.54	1.88 ± 0.75	0.63 ± 0.23	
	*IL-6*	1.90 ± 0.65	1.17 ± 0.52	0.34 ± 0.12	2.06 ± 0.77	2.29 ± 1.21	0.76 ± 0.36	
	*CD11b*	1.01 ± 0.07	1.02 ± 0.05	1.00 ± 0.06	1.04 ± 0.07	0.84 ± 0.08	0.99 ± 0.08	
	*CD45*	1.12 ± 0.22	1.09 ± 0.27	0.80 ± 0.16	1.28 ± 0.30	1.35 ± 0.38	0.79 ± 0.10	
	*TNF-α*	1.75 ± 0.69	1.17 ± 0.49	0.33 ± 0.11	1.93 ± 0.72	2.08 ± 1.07	1.23 ± 0.61	
*CA3*	*BDNF*	1.03 ± 0.11	0.96 ± 0.04	1.09 ± 0.06	1.09 ± 0.05	1.04 ± 0.07	0.94 ± 0.05	
	*IL-10*	0.87 ± 0.29	1.97 ± 0.76	4.57 ± 1.83	4.95 ± 2.04	6.54 ± 2.85	0.79 ± 0.19	
	*IL-1β*	1.02 ± 0.46	2.17 ± 0.76	2.74 ± 0.81	3.22 ± 1.30	3.53 ± 1.34	0.89 ± 0.11	
	*IL-6*	2.59 ± 1.35	3.47 ± 1.17	4.14 ± 1.38	6.36 ± 2.27	4.90 ± 2.17	1.41 ± 0.38	
	*CD11b*	1.01 ± 0.05	0.99 ± 0.04	1.26 ± 0.03	1.24 ± 0.07	1.13 ± 0.04	1.11 ± 0.03	Overall treatment effect (CBDV-100 and CBDV-20 > VEH)
	*CD45*	1.10 ± 0.21	1.14 ± 0.12	1.26 ± 0.15	1.51 ± 0.33	1.18 ± 0.22	0.80 ± 0.10	
	*TNF-α*	1.78 ± 0.80	1.74 ± 0.60	3.25 ± 1.12	3.12 ± 1.33	4.83 ± 2.26	0.59 ± 0.18	
*DG*	*BDNF*	1.01 ± 0.07	1.14 ± 0.06	1.05 ± 0.05	1.11 ± 0.07	1.05 ± 0.04	1.04 ± 0.05	
	*IL-10*	0.77 ± 0.17	1.43 ± 0.21	2.42 ± 0.87	0.77 ± 0.15	1.19 ± 0.24	1.08 ± 0.19	
	*IL-1β*	1.03 ± 0.11	1.10 ± 0.14	1.31 ± 0.28	0.95 ± 0.10	1.25 ± 0.24	1.19 ± 0.25	
	*IL-6*	1.11 ± 0.22	1.07 ± 0.19	1.71 ± 0.32	1.59 ± 0.30	1.97 ± 0.36	1.31 ± 0.23	
	*CD11b*	1.01 ± 0.07	0.97 ± 0.05	0.91 ± 0.06	0.95 ± 0.04	0.99 ± 0.04	1.13 ± 0.03	Overall treatment effect (CBDV-100 > CBDV-20)
	*CD45*	1.07 ± 0.16	1.14 ± 0.12	1.06 ± 0.06	1.19 ± 0.09	1.56 ± 0.15	1.35 ± 0.13	Overall treatment effect (CBDV100 > CBDV-20 and VEH)
	*TNF-α*	0.82 ± 0.09	1.37 ± 0.13	1.20 ± 0.15	0.97 ± 0.21	1.56 ± 0.36	1.44 ± 0.47	
*PFC*	*BDNF*	1.02 ± 0.08	1.00 ± 0.11	1.17 ± 0.11	1.26 ± 0.13	0.90 ± 0.07	0.92 ± 0.17	Overall treatment effect (CBDV-100 < CBDV-20)
	*IL-10*	1.36 ± 0.37	1.31 ± 0.35	1.45 ± 0.26	1.17 ± 0.46	1.34 ± 0.31	1.62 ± 0.75	
	*IL-1β*	1.05 ± 0.13	1.05 ± 0.21	1.07 ± 0.17	0.86 ± 0.12	1.09 ± 0.19	1.43 ± 0.29	
	*IL-6*	1.47 ± 0.47	1.47 ± 0.38	0.71 ± 0.12	1.07 ± 0.25	1.05 ± 0.17	3.15 ± 1.23	
	*CD11b*	1.12 ± 0.05	0.90 ± 0.05	1.11 ± 0.06	1.06 ± 0.08	1.01 ± 0.06	1.05 ± 0.12	
	*CD45*	1.23 ± 0.31	0.92 ± 0.14	1.22 ± 0.10	1.08 ± 0.11	1.44 ± 0.18	1.41 ± 0.25	
	*TNF-α*	1.39 ± 0.42	0.98 ± 0.22	0.70 ± 0.13	1.02 ± 0.36	1.38 ± 0.37	0.88 ± 0.09	

mRNA quantification was performed by RT-qPCR analysis. The relative level of expression was calculated with the comparative (2−ΔΔCT) method [51]. Data refer to bold change and are expressed as mean ± SEM. Sample size for each group was 7–9. Statistically significant differences are highlighted in yellow. CA1 = hippocampal CA1 subfield; CA3 = hippocampal CA3 subfield; DG = dentate gyrus; PFC = prefrontal cortex.

**Table 3:** Summary of the behavioral effects of the *Fmr1*-KO genotype and CBDV treatments.

Behavioral Domain	Test	Variable Measured	*ADULTS (Study 1)*			*JUVENILES (Study 2)*		
			Figures	KO Genotype Effect	CBDV Treatment Effect	Figures	KO Genotype Effect	CBDV Treatment Effect
Anxiety	EPM	% Time open arms	Figure S1	none	none	Figure S2	↓ anxiety	none
	OF	%Time center	Figure 2b	↓ anxiety in CBDV-100 only	CBDV-100 induces a KO phenotype	Figure 5b	↓ anxiety	none
Locomotor activity	EPM	total arm entries	Figure S1	none	none	Figure S3	↑ activity	none
	OF	Total distance moved	Figure 2a	↑ activity	none	Figure 5a	↑ activity	none
Learning & memory	OR	% OR index	Figure 2d	↓ object memory	↓ in WT mice, no rescue in KOs	Figure 5d	↓ object memory	↓ in WT mice, rescue in KOs
Social interest	3-COMP	% sociability index (trial 2)	Figure 3d	none	none	Figure 6d	none	none
		% social novelty index (trial 3)	Figure 3f	↓ social novelty preference	↓ in WT mice, no rescue in KOs	Figure 6f	↓ social memory	↓ in WT mice, rescue in KOs
	SI	Affiliation time	Figure 4a	↓ social interaction	↓ in WT mice, no rescue in KOs	Figure 7a	↓ social interaction	↓ in WT mice, rescue in KOs
Sensory responsiveness	ASR	startle body response	Figure 4b	↑ acoustic startle	rescue in KOs only with CBDV-20	Figure 7b	↑ acoustic startle	rescue in KOs

Significant genotype x treatment interactions are highlighted in blue. EPM = elevated plus maze; OF = open field; OR = object recognition; 3-COMP = 3-compartment test; SI = social interaction; ASR = acoustic startle response. ↑ increase. ↓ decrease.

## Data Availability

All original data are available upon reasonable request.

## Supplementary Materials

The following supporting information can be downloaded at: https://www.mdpi.com/article/10.3390/cells12151927/s1, Figure S1: Effects of adult subchronic CBDV treatment (20 or 100 mg/kg) in the elevated plus maze (Study 1); Figure S2: Effects of adult subchronic CBDV treatment on locomotor habituation in the open field (Study 1); Figure S3: Effects of juvenile chronic CBDV treatment (20 or 100 mg/kg) in the elevated plus maze (Study 2); Figure S4: Effects of juvenile chronic CBDV treatment on locomotor habituation in the open field (Study 2); Table S1: Primer Sequences used in RT-qPCR.
