# Supplementary material for: Early Administration of the Phytocannabinoid Cannabidivarin Prevents the Neurobehavioral Abnormalities Associated with the Fmr1-KO Mouse Model of Fragile X Syndrome
Source: Cells. 2023 Jul 25;12(15):1927. doi: 10.3390/cells12151927 (PMC10416983; doi:10.3390/cells12151927)
Supplement: Supplementary file 1 [file cells-12-01927-s001.zip › cells-2483450-supplementary.pdf]

Article

Early Administration of the Phytocannabinoid Cannabidivarin Prevents the Neurobehavioral Abnormalities Associated with the *Fmr1*-KO Mouse Model of Fragile X Syndrome

Marika Premoli [1,2,†], William Fyke [1,3,†], Luigi Bellocchio [4], Valerie Lemaire [1], Marie Wolley-Roberts [5], Bruno Bontempi [1,‡] and Susanna Pietropaolo [1,*,‡]

1 CNRS, EPHE, INCIA, UMR 5287, Univ. Bordeaux, 33000 Bordeaux, France
2 Department of Molecular and Translational Medicine, University of Brescia, 25123 Brescia, Italy
3 Graduate Program in Neural and Behavioral Science, SUNY Downstate Medical Center, 450 Clarkson Avenue, Brooklyn, NY 11203, USA
4 INSERM, U1215 NeuroCentre Magendie, Group Endocannabinoids and Neuroadaptation, University of Bordeaux, 33077 Bordeaux, France
5 Jazz Pharmaceuticals, Inc., Cambridge OX4 2RW, UK
* Correspondence: susanna.pietropaolo@u-bordeaux.fr
† Shared first authorship.
‡ Shared senior authorship.

Gene	GenBank ID	Forward Sequence (5′-3′)	Reverse Sequence (5′-3′)
Nono	NM_023144	CTGTCTGGTGCATTCCTGAACTAT	AGCTCTGAGTTCATTTTCCCATG
Sdha	NM_023281	TACAAAGTGCGGGTCGATGA	TGTTCCCCAAACGGCTTCT
Ywhaz	NM_011740	CTTGTGAGGCTGTGACACAAACA	CAAGAGTGTGCACGCAGACA
Tuba4a	NM_009447	CCACTTCCCCTTGGCTACCTA	CCACTGACAGCTGCTCATGGT
Gapdh	NM_008084	TCAAGAAGGTGGTGAAGCAG	TGGGAGTTGCTGTTGAAGTC
IL-1b	NM_008361	TCGCTCAGGGTCACAAGAAA	TCAGAGGCAAGGAGGAAAACAC
IL-6	NM_031168	TACTCGGCAAACCTAGTGCGT	ATTTTCTGACCACAGTGAGGAATG
IL-10	NM_010548	AGTTGTGAAGAAACTCATGGGTCT	TGCTGCAGGAATGATCATCAA
TNFα	NM_013693	GGCACTCCCCCAAAAGATG	GCCACAAGCAGGAATGAGAAG
ITGAM (CD11b)	NM_001082960	CTCATCACTGCTGGCCTATACAA	GCAGCTTCATTCATCATGTCCTT
PTPRC (CD45)	NM_011210	TGGGACAACGCAGACTCTCA	CTGCACAGCCATGTTCTTTCAT
BDNF	NM_007540	CCCGTCTGTACTTTACCCTTTGG	TGACTAGGGAAATGGGCTTAACA

Table S1. Primer sequences used in RT-qPCR to quantify mRNA.

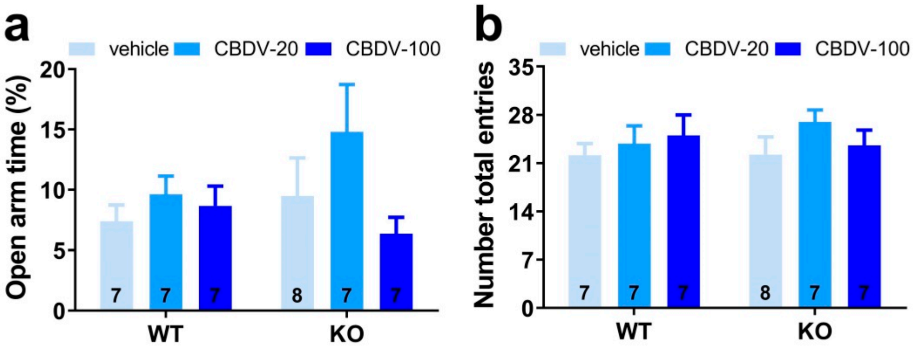

Figure S1. Effects of adult subchronic CBDV treatment (20 or 100 mg/kg) on anxiety as measured in the elevated plus maze (Study 1). Anxiety-like behavior (**a**) and locomotor activity (**b**) were assessed in WT and Fmr1-KO mice. Data are expressed as mean ± SEM. Numbers in histograms indicate sample size for each group.

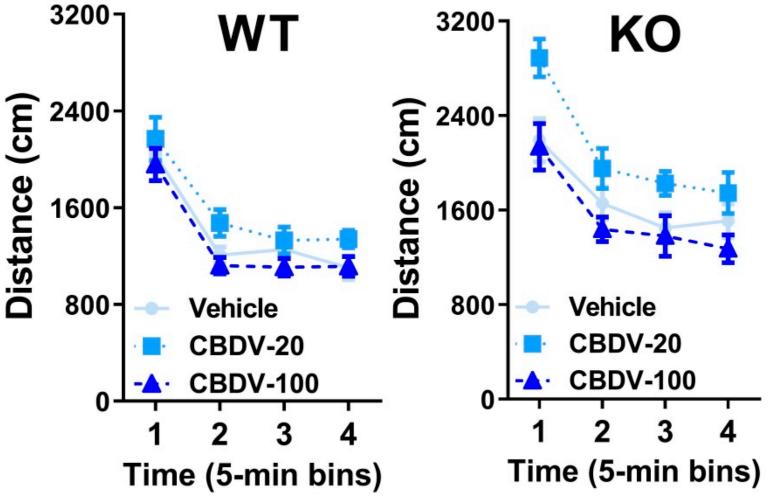

Figure S2. Effects of adult subchronic CBDV treatment on locomotor habituation in the open field (Study 1). Locomotor activity decreased during the 20-min habituation phase of the object recognition test in all experimental groups. Data are expressed as mean ± SEM. N=7-8 mice per group as in Figure 2a.

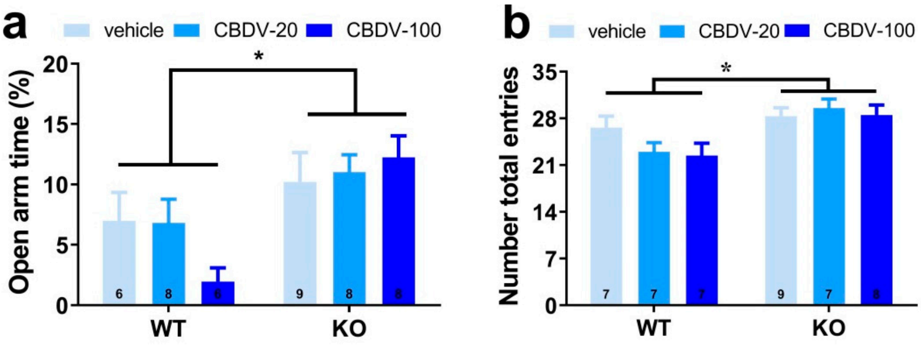

Figure S3. Effects of juvenile chronic CBDV treatment (20 or 100 mg/kg) on anxiety as measured in the elevated plus maze (Study 2). Anxiety-like behavior (**a**) and locomotor activity (**b**) were assessed in WT and Fmr1-KO mice. Data are expressed as mean ± SEM. Numbers in histograms indicate sample size for eachgroup. * indicates $p < 0.05$.

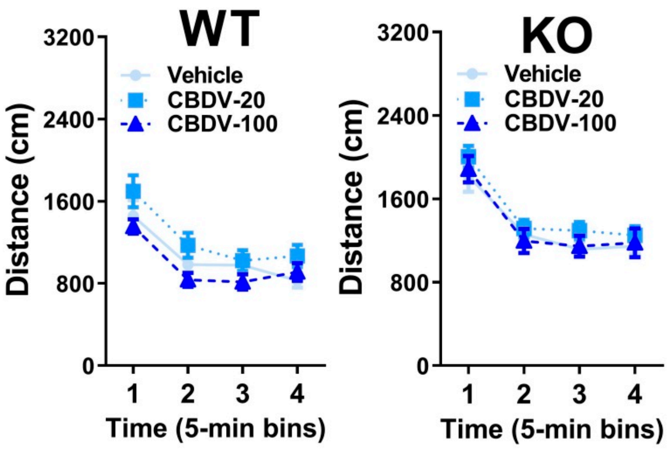

Figure S4. Effects of juvenile chronic CBDV treatment on locomotor habituation in the open field (Study 2). Locomotor activity decreased during the 20-min habituation phase of the object recognition test in all experimental groups. Data are expressed as mean ± SEM. N=7-8 mice per group as in Figure 5a.
